# WTAP-mediated m6A methylation of SOX2 affects lung adenocarcinoma malignancy via Wnt/β-catenin pathway

**DOI:** 10.3389/fgene.2026.1756241

**Published:** 2026-06-22

**Authors:** Na Li, Xiangbo Zeng, Hong Xie, Ye Fan, Shuying You

**Affiliations:** 1 Department of Respiratory and Critical Care Medicine, The Second People’s Hospital of Hunan Province (Brain Hospital of Hunan Province), Changsha, China; 2 Department of Pathology, The Second People’s Hospital of Hunan Province (Brain Hospital of Hunan Province), Changsha, China

**Keywords:** lung adenocarcinoma, m6A methylation, SOX2, Wnt/β-catenin, WTAP

## Abstract

**Background:**

Lung adenocarcinoma (LUAD) is one of the most common subtypes of lung cancer, characterized by high incidence and mortality rates. SOX2 is a transcription factor associated with the progression of various cancers. The role of the SOX2-Wnt/β-catenin signaling axis in LUAD has been previously reported; however, the upstream epitranscriptomic regulatory mechanisms governing SOX2, particularly the effects of m6A methylation on its expression, remain poorly elucidated. This study aims to investigate the role of SOX2 in the malignant progression of LUAD and its regulatory mechanisms, particularly its relationship with Wilms’ tumor 1-associating protein (WTAP).

**Methods:**

Bioinformatics analysis was used to analyze LUAD transcriptome data in the TCGA database, and to analyze the differential expression of SOX2 and WTAP. LUAD patient samples were collected for validation. The A549 cell line was used to construct SOX2 knockdown and WTAP overexpression models. The cell phenotype changes, signaling pathway protein expression, m6A methylation levels, and mRNA stability were evaluated using qRT-PCR, Western blotting, colony formation assay, Transwell assay, flow cytometry, m6A Dot blot assay, and RNA stability assay. Data analysis was conducted using SPSS software, with a significance level set at *P* < 0.05.

**Results:**

SOX2 was highly expressed in LUAD tissues, while WTAP expression was downregulated. Knockdown of SOX2 significantly inhibited the proliferation, migration, and invasion of LUAD cells and enhanced cell apoptosis (*P* < 0.05). WTAP reduced the stability of SOX2 mRNA through m6A modification, thereby inhibiting the activation of the Wnt/β-catenin signaling pathway and suppressing the malignant progression of LUAD (*P* < 0.05).

**Conclusion:**

This study reveals the oncogenic role of SOX2 in LUAD and its regulatory mechanism through the Wnt/β-catenin signaling pathway. It also elucidates the crucial role of WTAP in regulating SOX2 stability via m6A modification. These findings provide new potential therapeutic targets for LUAD, but further validation in animal models is needed.

## 1 Introduction

The latest global cancer epidemiology data reveal that lung cancer makes up approximately 12.4% of all new malignancy diagnoses and is the number one cause of cancer-related deaths (Bray et al., 2024). Lung adenocarcinoma (LUAD) is the most common among lung cancers, representing nearly 40% of all cases and thus being a major subtype encountered in clinical settings (Duma et al., 2019; Succony et al., 2021). Surgical resection remains the cornerstone of LUAD treatment, supported by chemotherapy and targeted therapies to maximize therapeutic benefits (Bian et al., 2023; Wang Y. et al., 2021; Wang et al., 2023). However, complications such as drug resistance and adverse reactions are severely diminishing the effectiveness of these treatments (Zuo et al., 2023; Yu et al., 2023). As such, a deeper exploration of molecular pathways involved in LUAD and the identification of biomarkers with diagnostic and therapeutic value are essential for enhancing clinical strategies.

Wnt/β-catenin signaling is critical for regulating embryonic development, stem cell self-renewal, and the maintenance of tissue homeostasis in adult organisms (Liu et al., 2022; Zhang and Wang, 2020). Aberrant Wnt/β-catenin signaling activation is intricately linked to the development of various malignancies. This pathway modulates downstream target gene expressions, thereby influencing cell apoptosis, differentiation, migration, proliferation, and invasion (Salik et al., 2020; Soleas et al., 2020; Choi et al., 2020). Additionally, it substantially contributes to the modulation of immunosuppressive microenvironments and facilitates immune evasion (Zhou et al., 2022; Mortezaee, 2024). Despite extensive research on Wnt/β-catenin signaling in tumorigenesis, upstream mechanisms of its abnormal activation in LUAD remain unclear.

SOX2, a prominent member of the SOX family of transcription factors (TFs), is crucial for the development of various tissues and organs, especially in regulating stem cell self-renewal and pluripotency (Novak et al., 2020; Zhu et al., 2021). Recent investigations into SOX2 have revealed its critical involvement in the pathogenesis of multiple cancers, encompassing lung cancer (Rudin et al., 2012; Ying et al., 2016; Nakatsugawa et al., 2011), cervical cancer (Yuan et al., 2021), oral squamous cell carcinoma (Sacco et al., 2023), and bladder cancer (Chiu et al., 2020). The overexpression of SOX2 appears to fuel cell proliferation, enable cancer cells to bypass apoptosis, and boost their invasive potential. In non-small cell lung cancer, upregulation of SOX2 recruits regulatory T cells, thereby modulating immune checkpoint blockade therapy (Torres-Mejia et al., 2025). SOX2 can serve as a downstream target of epidermal growth factor receptor (EGFR) signaling and plays an important role in the self-renewal and expansion of progenitor cells (Karachaliou et al., 2013). Although SOX2 protects lung cancer cells from ferroptosis by upregulating SLC7A11, contributing to tumor progression (Wang X. et al., 2021), the broader molecular mechanisms by which SOX2 influences LUAD are still under investigation. Clarifying these pathways could reveal new therapeutic opportunities for LUAD.

Wilms’ tumor 1-associating protein (WTAP), acting as a methyltransferase, is capable of regulating m6A modification of mRNA (Wang et al., 2020). Specifically, WTAP binds to methyltransferase-like 3 (METTL3) in the nucleus to form the methyltransferase-like 3-methyltransferase-like 14 (METTL14)-WTAP (MMW) complex, which is localized in the nucleus and thereby modifies m6A (Fan et al., 2022). Moreover, elevated levels of WTAP are associated with poor prognosis in NSCLC (Cheng et al., 2022). This study demonstrates that SOX2 is markedly elevated in LUAD tissues and cells. SOX2 knockout is shown to effectively curb the malignant phenotype of LUAD cells by inhibiting the Wnt/β-catenin signaling. It was also found that WTAP, an m6A methyltransferase-associated protein, could mediate m6A modification of SOX2, destabilize its mRNA, and accelerate its degradation, ultimately influencing tumor progression. These findings elucidate mechanisms of SOX2 in LUAD and offer new potential therapeutic targets.

## 2 Materials and methods

### 2.1 Bioinformatics

LUAD transcriptomic data were obtained from TCGA (https://portal.gdc.cancer.gov/). By using R software, expression differences of SOX2 and WTAP in LUAD tissues (n = 347) and adjacent normal tissues (n = 483) were analyzed, with screening criteria set at *P* < 0.05 and |log2FC|>2. The RM2Target database (http://rm2target.canceromics.org/) was utilized to predict m6A methylation modification genes related to SOX2 and to identify potential motif sites involved in SOX2 m6A modification. Systematic scanning of the full-length SOX2 mRNA sequence (NM_003106.4) was performed based on the DRACH motif (5′-DRACH-3′, D = [A/G/U], R = [A/G], H = [A/C/U]). A total of 43 potential m6A modification sites were identified. According to the well-documented preferential distribution characteristics of m6A sites, confidence scoring was conducted for each candidate site. High-frequency motifs such as GGACC and GGACT were assigned higher weights, while additional positional scores were given to sites near the stop codon (the 3′ terminal of CDS) and the proximal region of the 3′UTR. In total, 19 high-confidence sites with a score ≥0.85 were screened out. Among them, the highest-confidence m6A locus in the 3′UTR was identified as A1701 (GGACC, 3′UTR +311 nt).

### 2.2 Clinical sample retrieval

This study recruited LUAD patients who received treatment at the Second People’s Hospital of Hunan Province (Brain Hospital of Hunan Province) between January 2024 and November 2024. Tumors and corresponding adjacent normal tissues were obtained from these patients. To ensure sample quality and diagnostic accuracy, all samples were independently reviewed by two pathologists. All participants provided informed consent to donate tissue samples and authorize the use of their clinical data for research analysis. The study protocol was approved by the Ethics Review Committee of the Second People’s Hospital of Hunan Province (Brain Hospital of Hunan Province), approval number: (2021K021), and strictly followed ethical guidelines to ensure compliance and protect patients’ rights.

### 2.3 Cell culture

For this study, normal human bronchial epithelial cells (BEAS-2B, BNCC359274) and two LUAD cell lines, A549 (BNCC337696) and HCC827 (BNCC358193), were all purchased from BNCC (China). Cells were cultured under the following conditions: BEAS-2B cells were cultured in DMEM-H medium with 10% FBS; A549 cells were maintained in F-12K medium containing 10% FBS; and HCC827 cells were cultured in RPMI-1640 medium supplemented with 10% FBS. All media were obtained from BNCC (China) and included 1% penicillin-streptomycin solution (Procell, China). The cells were cultured in a sterile incubator at 37 °C with 5% CO_2_.

### 2.4 Cell transfection

The siRNA targeting SOX2 (si-SOX2), overexpression of WTAP plasmids pcDNA3.1 (oe-WTAP) and overexpression of SOX2 plasmids pcDNA3.1 (oe-SOX2), alongside their negative controls (si-NC and oe-NC), were provided by RiboBio (China). The transfection process into A549 cells using Lipofectamine 2000 (Thermo Fisher, USA) was carried out as follows: A549 cells were cultured until they reached 70%–90% confluence. Lipofectamine® reagent and siRNA or DNA plasmids were then diluted separately in Opti-MEM® medium (Thermo Fisher, USA), mixed in a 1:1 ratio, and incubated at room temperature for 5 min. The mixture was subsequently added to the cell culture and incubated at 37 °C for 1–3 days. After the transfection efficiency was validated, the cells were used for subsequent studies.

### 2.5 qRT-PCR

Total RNA was extracted from A549 cells utilizing TRIzol reagent (Invitrogen, USA). The RNA was then reverse-transcribed into cDNA with the aid of the PrimeScript RT Master Mix (Takara, Japan). qRT-PCR was done on the ABI7500 system (Thermo Fisher, USA), with each sample run in triplicate. GAPDH was utilized as the endogenous reference gene, and the 2^−ΔΔCT^ method was applied to compute relative expressions. Primer sequences for all targets are provided in Table 1 for reference.

**TABLE 1:** Primer sequences.

Gene	Primer sequence
WTAP	Forward primer: 5′-GCT​TCT​GCC​TGG​AGA​GGA​TT-3’; reverse primer: 5′-GTG​TAC​TTG​CCC​TCC​AAA​GC-3′
SOX2	Forward primer: 5′-AAC​CAG​CGC​ATG​GAC​AGT​TA-3’; reverse primer: 5′-CGA​GCT​GGT​CAT​GGA​GTT​GT-3′
β-catenin	Forward primer: 5′-AAT​CAG​CTG​GCC​TGG​TTT​GA-3’; reverse primer: 5′-TCC​CAC​CCT​ACC​AAC​CAA​GT-3′
GAPDH	Forward primer: 5′-AAT​GGG​CAG​CCG​TTA​GGA​AA-3’; reverse primer: 5′-GCG​CCC​AAT​ACG​ACC​AAA​TC-3′

### 2.6 Western blotting

Cells were first rinsed with pre-cooled PBS buffer following different treatments and then lysed at 4 °C for 30 min. Equal amounts of protein samples were separated by SDS-PAGE and transferred to PVDF membranes (Millipore, USA). The membranes were sealed with 5% skim milk for 45 min, incubated with primary antibodies at 4 °C for 12 h, and subsequently reacted with HRP-conjugated secondary antibodies for 1 h at room temperature. After rinsing with PBST, membranes were visualized with ECL chemiluminescent reagent (Thermo Fisher, USA) and imaged utilizing the iBright imaging system (Thermo Fisher, USA) for analysis. Antibodies are listed in Table 2.

**TABLE 2:** Antibodies.

Antibody	Catalog number and supplier
WTAP	ab195380; Abcam (UK)
SOX2	ab92494; Abcam (UK)
c-MYC	ab32072; Abcam (UK)
β-catenin	ab32572; Abcam (UK)
goat anti-rabbit IgG	ab6721; Abcam (UK)
GAPDH	ab181602; Abcam (UK)

### 2.7 Transwell

Following a 24-h culture, cells were harvested using trypsin (Beyotime, China). For the migration assay, 2 × 10^4^ cells were suspended in 200 µL of serum-free medium and placed into the upper chamber of a Transwell insert (Corning, USA). Lower chamber contained 700 µL of F-12K medium (BNCC, China) plus 10% FBS. After 24 h, non-migrated cells were removed, and the migrated cells were fixed with 75% ethanol and stained with 0.5% crystal violet (Beyotime, China). Cells were observed under a microscope (Carl Zeiss, Germany) at 10× magnification. For the invasion assay, the upper chamber of the Transwell insert was coated with 100 µL of Matrigel matrix (BD Biosciences, USA) and incubated for 2 h at 37 °C. Cell density was adjusted to 2 × 10^5^ cells/mL, and 200 µL of the cell suspension was added to the upper chamber, with 700 µL of F-12K medium plus 10% FBS in the lower chamber. Cells were incubated for 24 h, and other steps were identical to those in the migration assay.

### 2.8 Colony formation assay

About 100 cells/well were seeded in a 12-well plate. Cells were regularly monitored for morphological changes and the medium was refreshed periodically. After 10–14 days, when visible colonies formed at the bottom of the wells, the wells were rinsed twice with PBS. Cells were then fixed with 4% paraformaldehyde (Beyotime, China) for 15 min and stained with 0.5% crystal violet (Beyotime, China) for 30 min. Finally, colonies were counted.

### 2.9 Cell apoptosis detection

Cells were digested using 0.25% trypsin (Beyotime, China) and harvested. In the Annexin V-FITC/PI double-staining experiment, A549 cells were collected with PBS and resuspended in 100 µL of binding buffer. Then, 10 µL of Annexin V-FITC and PI staining solution was added to the cell suspension, and the mixture was incubated at room temperature for 15 min in the dark. Cell apoptosis was assessed utilizing an Agilent flow cytometer (USA), and the data were processed using Agilent’s software (USA).

### 2.10 Cell cycle analysis

After cell digesting with 0.25% trypsin (Beyotime, China), PI/RNase staining was performed using a cell cycle and apoptosis detection kit (Beyotime, China). Specifically, the treated A549 cells were collected with PBS and fixed overnight at −20 °C with pre-cooled 70% ethanol. The next day, the ethanol was discarded by centrifugation, and the cells were washed twice with PBS and resuspended in 500 μL PI/RNase staining solution, followed by incubation at 37 °C in the dark for 30 min. Finally, cell cycle distribution was detected using a flow cytometer (Agilent, USA), and the detection data were processed with analysis software (Agilent, USA).

### 2.11 m6A dot-blot

TRIzol reagent (Invitrogen, USA) was used to isolate RNA from A549 cells. After quantification and denaturation, RNA was transferred to a Hybond N+ membrane (GE Healthcare, USA) and cross-linked three times using the automatic cross-linking mode. The membrane was stained with a sodium acetate solution containing 0.02% methylene blue (Sigma, USA) after UV cross-linking. It was then washed with 1× PBST, sealed with 5% skim milk for 1 h, and incubated with an m6A-specific antibody (202003, Synaptic Systems, Germany) overnight at 4 °C. The following day, the membrane was treated with an HRP-conjugated secondary antibody (ab6721, Abcam, UK) and visualized by Amersham ECL Prime (GE Healthcare, USA).

### 2.12 MeRIP-qPCR

Total m6A modification levels were detected using an N6-methylated RNA immunoprecipitation (MeRIP) kit (RIBOBIO, China). All experimental procedures were strictly performed in accordance with the manufacturer’s instructions. Briefly, total RNA was extracted with TRIzol reagent. The isolated RNA was mixed with fragmentation buffer and incubated at 94 °C for 3 min to generate fragmented RNA. A total of 25 μL Protein A/G magnetic beads were pre-washed and then incubated with 5 μg of anti-m6A antibody (ab208577; Abcam) for conjugation. The antibody-bead complexes were further incubated with fragmented RNA to capture m6A-modified transcripts. After magnetic separation and thorough washing to remove unbound RNA, the immunoprecipitated RNA was eluted from the beads and purified. Finally, the enrichment of m6A-modified SOX2 mRNA was quantified by qRT-PCR.

### 2.13 RIP

The RIP assay was conducted using the Imprint RNA Immunoprecipitation Kit (Sigma-Aldrich, USA), and all operations were strictly implemented in accordance with the manufacturer’s instructions. In brief, A549 cells were collected and lysed with RIP lysis buffer to obtain cell lysates. The lysates were incubated with 1 μg of WTAP antibody (ab195380; Abcam, UK) at 4 °C for 3 h to form WTAP-RNA immune complexes. Afterwards, magnetic beads were applied to capture and precipitate the complexes. After washing to eliminate non-specific binding RNAs, the bound RNAs were extracted. Finally, the enrichment of SOX2 RNA bound to WTAP protein was measured via qRT-PCR.

### 2.14 RNA stability analysis

Cells were grown to 70%–80% confluence after different treatments. Actinomycin D (5 μg/mL, MCE, USA) was added to block RNA synthesis, and cells were incubated for 0, 2, 4, and 6 h. RNA was extracted at each time point and analyzed by qRT-PCR. Relative abundance of mRNA was evaluated by the 2^−ΔΔCT^ method, with Ct values at 0 h as the baseline. The primer sequences are detailed in Table 1.

### 2.15 Statistical analysis

Experiments were independently repeated 3 times, and results are expressed as mean ± SEM. Data analysis was done on SPSS 21.0 (IBM, USA), with *t*-tests for comparisons between two groups and one-way ANOVA for multiple groups. Data visualization was completed using GraphPad Prism 8 (GraphPad, USA). Significance level was set at *P* < 0.05.

## 3 Results

### 3.1 Upregulation of SOX2 in LUAD

**FIGURE 1:**
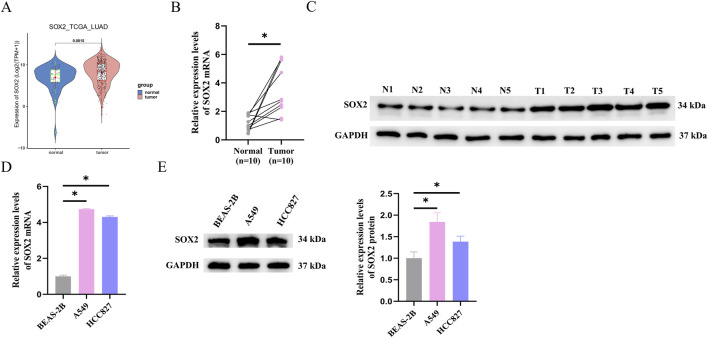
Upregulation of SOX2 in LUAD. **(A)** Differential expression of SOX2 (log2 (TPM+1)) in LUAD was analyzed using the TCGA-LUAD dataset; **(B)** qRT-PCR was conducted to assess the mRNA expression levels of SOX2 in LUAD tissues and adjacent normal tissues; **(C)** Western blotting was conducted to assess the protein expression levels of SOX2 in LUAD tissues and adjacent normal tissues; **(D)** The mRNA expression of SOX2 was measured in human normal bronchial epithelial cells (BEAS-2B) and LUAD cell lines (A549, HCC827) by qRT-PCR. **(E)** The protein expression of SOX2 was measured in human normal bronchial epithelial cells (BEAS-2B) and LUAD cell lines (A549, HCC827) by Western blotting. *P < 0.05.

SOX2 levels in LUAD were examined by leveraging TCGA-LUAD, which revealed that SOX2 mRNA was obviously more abundant in LUAD tissues than in normal lung tissues (Figure 1A). To verify the bioinformatics results, qRT-PCR was performed on 10 pairs of LUAD and adjacent non-cancerous tissues. qRT-PCR confirmed that SOX2 expression was considerably elevated in LUAD tissues compared to adjacent tissues (Figure 1B). Western blotting analysis of 5 paired LUAD and adjacent normal tissues further revealed that SOX2 protein expression was significantly upregulated in LUAD tissues (Figure 1C). Cellular assays further elucidated that SOX2 expression was markedly elevated in A549 and HCC827 cells compared to BEAS-2B cells (Figures 1D,E). This consistent pattern solidifies the evidence of the aberrant overexpression of in LUAD.

### 3.2 SOX2 knockdown attenuates LUAD malignancy

**FIGURE 2:**
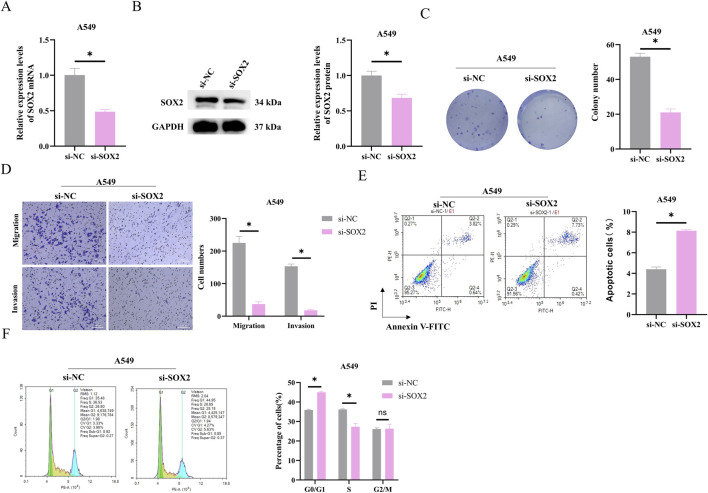
SOX2 knockdown attenuates LUAD malignancy. A549 cells were stratified into two groups: si-NC and si-SOX2. **(A)** Transfection efficiency was confirmed by qRT-PCR in each group; **(B)** Western blotting was used to detect the protein expression of SOX2. **(C)** Colony formation assays were used to evaluate cell proliferation in each group; **(D)** Migration and invasion were analyzed using Transwell assays in each group; **(E)** Apoptosis was detected by flow cytometry in each group. **(F)** Cell cycle was detected by flow cytometry in each group. *P < 0.05.

To understand the role of SOX2 overexpression on LUAD malignancy, siRNA was used to knock down SOX2 in A549 and HCC827 cells, which have high SOX2 expression. qRT-PCR confirmed the knockdown efficiency (Figure 2A) (Supplementary Figure S1A). The results of Western blotting indicated that the knockdown of SOX2 significantly reduced the protein expression in A549 and HCC827 cells (Figure 2B) (Supplementary Figure S1B). In colony formation assays, SOX2 knockdown was observed, capable of curbing the proliferation of A549 and HCC827 cells (Figure 2C) (Supplementary Figure S1C). Transwell assays further unveiled that SOX2 knockdown obviously weakened the migratory and invasive capabilities of A549 and HCC827 cells (Figure 2D) (Supplementary Figure S1D). Flow cytometry analysis also indicated an appreciably higher apoptosis rate in A549 and HCC827 cells following SOX2 knockdown (Figure 2E) (Supplementary Figure S1E). Existing literature has shown that the knockdown of SOX2 expression inhibits cell cycle progression by arresting cells in the G0/G1 phase and that the knockdown of SOX2 suppresses the expression of cyclin D1 (Chen et al., 2020; Moura et al., 2016). Flow cytometry results also indicated that the knockdown of SOX2 arrested the cell cycle of A549 and HCC827 cells in the G0/G1 phase (Figure 2F) (Supplementary Figure S1F). Overall, SOX2 knockdown effectively inhibited the malignant behaviors of LUAD cells.

### 3.3 SOX2 promotes LUAD progression by activating the Wnt/β-catenin pathway

**FIGURE 3:**
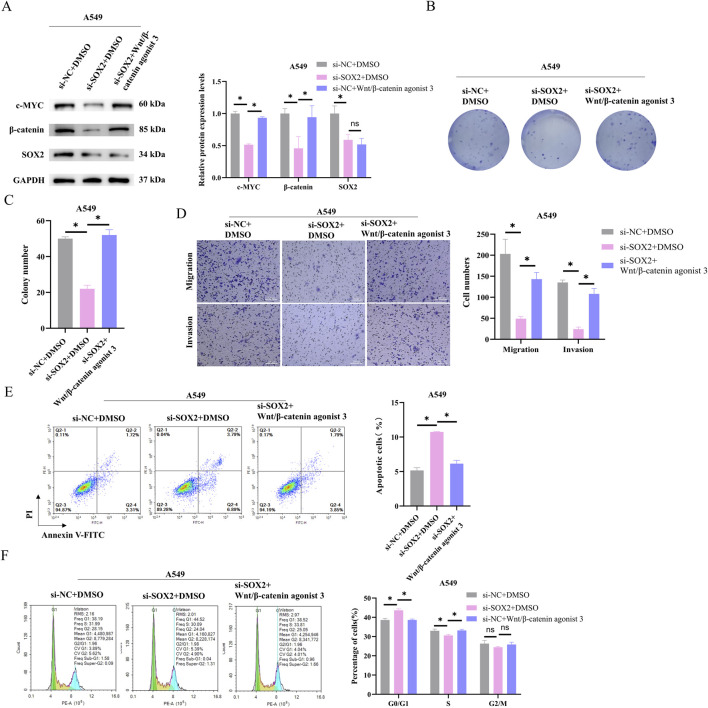
SOX2 affects LUAD progression through the Wnt/β-catenin pathway. A549 cells were grouped into four categories: si-NC + DMSO, si-SOX2 + DMSO, and si-SOX2 + Wnt/β-catenin agonist 3. **(A)** Western blotting was used to detect the protein expression of SOX2, β-catenin, and c-Myc; **(B,C)**: Proliferation was evaluated by colony formation assays in each group; **(D)** Migration and invasion were analyzed using Transwell in each group; **(E)** Apoptosis was detected by flow cytometry in each group. **(F)** Cell cycle was detected by flow cytometry in each group. *P < 0.05.

With the evidence that SOX2 was upregulated in LUAD and enhanced the malignant phenotype of LUAD cells, the potential role of Wnt/β-catenin signaling was further explored. Although prior research has shown that SOX2 modulates Wnt/β-catenin signaling (Shi et al., 2022; Mukherjee et al., 2022), it is still uncertain whether the effects of SOX2 on LUAD progression are mediated through this pathway. To address this gap, experiments were conducted by knocking down SOX2 and treating cells with the Wnt pathway activator Wnt/β-catenin agonist 3 (MCE, USA). The experimental design includes three groups: treated with DMSO after transfection with negative control siRNA (si-NC + DMSO), treated with DMSO after transfection with SOX2-targeting siRNA (si-SOX2+DMSO), and treated with a Wnt pathway activator after transfection with SOX2-targeting siRNA (si-SOX2+Wnt/β-catenin agonist 3). SOX2 knockdown markedly inhibited Wnt/β-catenin pathway activity and malignant cellular phenotypes, whereas exogenous activation of the Wnt/β-catenin pathway partially mimicked the functional effects of SOX2. These findings indicate that SOX2 facilitates LUAD progression via activating the Wnt/β-catenin signaling pathway (Figure 3A). Wnt/β-catenin agonist 3 (MedChemExpress) is a selective GSK-3β inhibitor. Mechanistically, it represses the kinase activity of GSK-3β and blocks the phosphorylation of β-catenin at the Ser33/37/Thr41 residues. This blockade prevents the ubiquitination and proteasomal degradation of β-catenin, thereby promoting its cytoplasmic accumulation and subsequent nuclear translocation (Yu et al., 2019; Jin et al., 2022). To further validate this regulatory mechanism, we detected β-catenin mRNA levels by qPCR. The results showed that SOX2 knockdown significantly reduced β-catenin transcription, while treatment with Wnt/β-catenin agonist 3 exerted no obvious influence on β-catenin mRNA expression (Supplementary Figure S2A). Colony formation and Transwell assays reported that SOX2 knockdown drastically curbed cell proliferation, migration, and invasion (Figures 3B–D). However, these effects were effectively reversed by treatment with Wnt/β-catenin agonist 3. Moreover, flow cytometry showed that SOX2 knockdown promoted cell apoptosis, an effect that was reversed by Wnt/β-catenin signaling activation (Figure 3E). It has been reported that Wnt pathway activators can promote the transition of osteosarcoma cells from the G1/G0 phase to the S phase (Wei et al., 2024). Flow cytometry indicated that SOX2 knockdown induced cell cycle arrest in the G0/G1 phase, which was reversed by the activation of the Wnt/β-catenin pathway (Figure 3F). SOX2 directly activated Wnt/β-catenin signaling, evidenced by increased β-catenin and c-Myc expression upon SOX2 overexpression. Functionally, pharmacological inhibition of Wnt/β-catenin signaling abrogated the pro-tumorigenic effects of SOX2 overexpression, confirming that SOX2 promotes LUAD progression through Wnt/β-catenin pathway activation.

### 3.4 WTAP-mediated m6A methylation promotes SOX2 mRNA decay

**FIGURE 4:**
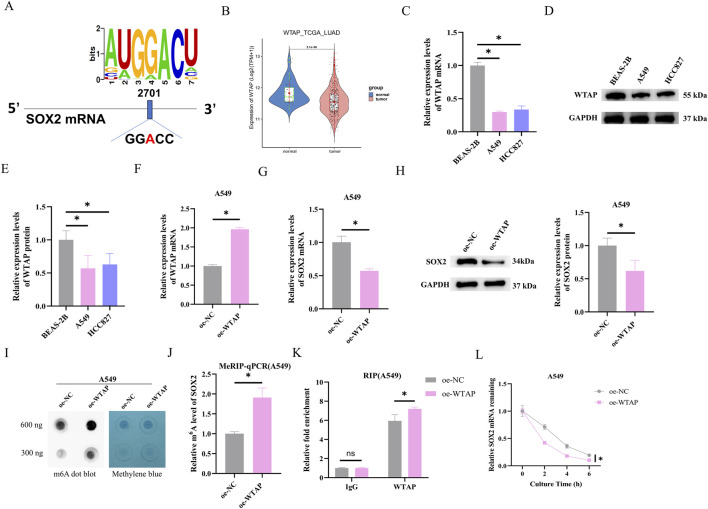
WTAP-mediated m^6^A methylation promotes SOX2 mRNA decay. **(A)** m^6^A modification sites of SOX2 targeted by WTAP were predicted using bioinformatics tools; **(B)** Differential expression analysis of WTAP (log2 (TPM+1)) in LUAD was conducted based on the TCGA-LUAD dataset; **(C)** qRT-PCR was used to measure the mRNA expression levels of WTAP in human normal lung epithelial cells (BEAS-2B) and LUAD cells (A549, HCC827). **(D,E)** Western blotting was used to measure the protein expression levels of WTAP in human normal lung epithelial cells (BEAS-2B) and LUAD cells (A549, HCC827). A549 cells were assigned to two groups: oe-NC and oe-WTAP. **(F)** The mRNA expression of WTAP in each group was detected by qRT-PCR; **(G)** The mRNA expression levels of SOX2 in each group were measured by qRT-PCR; **(H)** Western blotting was used to measure the protein expression levels of SOX2. **(I)** m^6^A methylation levels in each group were detected by dot-blot assays; **(J)** The mRNA expression of SOX2 in each group was detected by MeRIP; **(K)** The mRNA expression of SOX2 in each group was detected by RIP; **(L)** The mRNA stability of SOX2 in each group was assessed by qRT-PCR. *P < 0.05.

New research illuminates the significant impact of methylation regulators on the progression of LUAD, from its inception to advanced stages (Ding et al., 2024). To identify the factors mediating SOX2 m6A methylation in LUAD, online prediction tools were used to identify WTAP as a likely candidate (Figure 4A). Bioinformatics analysis revealed that WTAP was downregulated in LUAD tissues (Figure 4B). To confirm these findings, qRT-PCR and Western blotting measured WTAP mRNA and protein expression in BEAS-2B and LUAD cells (A549, HCC827). The results were consistent with our bioinformatics analysis, showing that WTAP was tangibly downregulated in LUAD cells (Figures 4C–E). To examine the effects of WTAP on SOX2 mRNA expression, A549 cells were transfected with empty plasmids (oe-NC) or WTAP-overexpressing plasmids (oe-WTAP) and confirmed the transfection efficiency by qRT-PCR (Figure 4F). qRT-PCR data unraveled that overexpressing WTAP dramatically lowered SOX2 mRNA levels (Figure 4G). Western blotting results confirmed that WTAP overexpression suppressed the protein expression of SOX2 (Figure 4H). Additionally, dot-blot assays were used to measure the m6A methylation levels in the cells and found that WTAP overexpression led to an increase in m6A methylation levels (Figure 4I). Meanwhile, the MeRIP assay was performed to verify the regulatory effect of WTAP on the m6A methylation level of SOX2. The results demonstrated that WTAP overexpression increased the mRNA enrichment of SOX2, indicating a significant elevation in its m6A methylation level (Figure 4J). In addition, RIP analysis revealed that WTAP overexpression notably enhanced the enrichment of SOX2 mRNA compared with the control group (Figure 4K). To assess the effects of WTAP on SOX2 mRNA stability, A549 cells were transfected with oe-NC or oe-WTAP plasmids with 5 μg/mL actinomycin D (MCE, USA) to block new RNA synthesis and measured SOX2 mRNA levels by qRT-PCR. Overexpressing WTAP decreased the stability of SOX2 mRNA (Figure 4L). Collectively, these findings suggest that in LUAD cells, WTAP could mediate the m6A methylation of SOX2 and promote its mRNA decay.

### 3.5 WTAP affects the malignant progression of LUAD via the SOX2-mediated Wnt/β-catenin pathway

**FIGURE 5:**
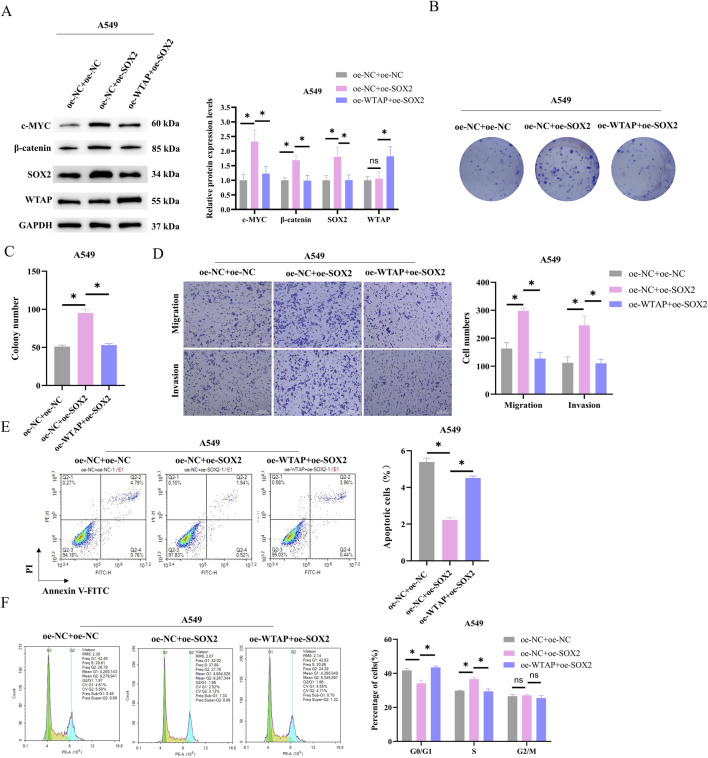
WTAP affects the malignant progression of LUAD through the SOX2-mediated Wnt/β-catenin pathway. A549 cells were grouped into three categories: oe-NC + oe-NC, oe-NC + oe-SOX2, and oe-WTAP + oe-SOX2. **(A)** Western blotting was used to detect the protein expression levels of SOX2, β-catenin, and c-Myc in each group; **(B,C)**: Colony formation assays were performed to evaluate the proliferative capacity of each group; **(D)** Migration and invasion abilities were analyzed using Transwell assays in each group; **(E)** Apoptosis was detected by flow cytometry in each group. **(F)** Cell cycle was detected by flow cytometry in each group. *P < 0.05.

To investigate whether WTAP affects the malignant phenotype of LUAD cells by modulating SOX2 and Wnt/β-catenin signaling, SOX2 and WTAP were designed to be overexpressed in A549 cells. The groups were configured as follows: the group transfected with empty control plasmids and empty control plasmids (oe-NC + oe-NC), the group transfected with empty control plasmids and SOX2 overexpression plasmids (oe-NC + oe-SOX2), and the group transfected with WTAP overexpression plasmids and SOX2 overexpression plasmids (oe-WTAP + oe-SOX2). By using Western blotting, protein expression associated with Wnt/β-catenin signaling was examined. The results (Figure 5A) indicated that overexpressing SOX2 strikingly increased SOX2, β-catenin, and c-Myc protein levels. However, overexpressing WTAP attenuated the upregulatory effect of SOX2 overexpression on these proteins. Overexpressing WTAP in cells already overexpressing SOX2 resulted in a substantial reduction in cell proliferation, migration, and invasion, as evidenced by colony formation and Transwell assays (Figures 5B–D). Flow cytometry analysis further indicated that WTAP overexpression effectively counteracted the apoptosis resistance induced by SOX2 overexpression (Figure 5E). Overexpression of WTAP significantly inhibited SOX2-induced cell cycle progression (Figure 5F). Overall, WTAP could modulate the malignant phenotype of LUAD cells by modulating SOX2-mediated Wnt/β-catenin signaling.

## 4 Discussion

SOX2, a widely investigated TF, has been linked to the progression of multiple cancers (Novak et al., 2020). Despite existing studies on the involvement of SOX2 in LUAD, the specific mechanisms by which SOX2 contributes to LUAD development have not been clarified. The current research indicates that SOX2 is upregulated in LUAD tissues and cells, with its expression levels being strongly correlated with tumor cell malignancy. The critical mechanism in LUAD was also uncovered: WTAP hastens the decay of SOX2 mRNA via m6A methylation, thereby inhibiting activation of Wnt/β-catenin signaling and curbing tumor cell proliferation. This discovery not only underscores the pivotal role of SOX2 in LUAD but also elucidates how the WTAP/SOX2 axis governs disease progression. Our study uncovers new dimensions of the molecular architecture of LUAD and suggests new avenues for targeted therapies based on m6A methylation.

Oncology studies have consistently shown that SOX2 is upregulated in various cancers, including gastric cancer (Cao et al., 2024), oral squamous cell carcinoma (Sacco et al., 2023), and ovarian cancer (Dorsett et al., 2019). This overexpression has been linked to cancer progression and serves as a prognostic biomarker for multiple malignancies (Yuan et al., 2021; Liu et al., 2021). This research has revealed a critical role for SOX2 in LUAD, where its elevated expression is linked to more aggressive cellular proliferation, migration and invasiveness, and enhanced resistance to apoptosis. Despite these observations, the precise regulatory pathways through which SOX2 exerts its oncogenic effects are still being explored. β-catenin has emerged as a possible molecular target of SOX2 (Shi et al., 2022). A prior study unveiled that SOX2 can promote breast cancer progression via activation of the Wnt/β-catenin pathway (Meng et al., 2020). Similarly, SOX2 could also activate Wnt/β-catenin signaling in LUAD, thereby contributing to its malignant progression, which adds to the growing body of evidence on SOX2-mediated Wnt/β-catenin signaling in LUAD and advances our understanding of the mechanisms driving LUAD development. However, our findings are inconsistent with the inhibitory effect of SOX2 reported by He et al. (2017) in cisplatin-resistant cells. Such a discrepancy may be attributed to the cell context-dependent function of SOX2. Under chemoresistant conditions, SOX2 may maintain cancer stem cell properties and restrain Wnt signaling activity by upregulating GSK3β. In contrast, under conventional proliferative conditions, SOX2 acts as a transcription factor to directly activate Wnt target genes and facilitate tumor progression. Notably, the upstream regulatory mechanism of SOX2 was not investigated in He et al’s study. The newly identified WTAP-m6A-SOX2-Wnt axis in our study provides a novel perspective for understanding the dynamic regulation of SOX2 expression.

m6A modification, a frequent chemical alteration found on mRNA in eukaryotes, is now recognized as a key factor influencing tumor formation and progression (Wiener and Schwartz, 2021). m6A modification regulates RNA function and expression levels by influencing different steps of RNA metabolism (An and Duan, 2022). In this process, three key enzyme classes—writers, readers, and erasers—participate in dynamic regulation of m6A modification (Cusenza et al., 2023). WTAP, a core component of the mRNA methyltransferase complex and an m6A writer, is responsible for adding methyl groups to specific adenosine residues to control m6A modification (Wang et al., 2020). WTAP-mediated m6A modification impacts the progression of a range of cancers by altering mRNA stability (Fan et al., 2022). This mechanism is particularly evident in hepatocellular carcinoma, where the modification promotes tumor growth via the HuR-ETS1-p21/p27 axis (Chen et al., 2019). The present study discovers that WTAP is downregulated in LUAD, which impairs its ability to modify SOX2 through m6A methylation. This disruption leads to increased stability of SOX2 mRNA and further contributes to the progression of LUAD. These findings elucidate interaction between WTAP and SOX2 and the oncogenic role of the WTAP/SOX2 axis in LUAD, offering a novel perspective on the molecular underpinnings of this cancer.

Our investigation has pinpointed the overexpression of SOX2 in LUAD and uncovered how WTAP regulates SOX2 stability via m6A methylation, thereby influencing Wnt/β-catenin signaling and fueling cancer progression. While these insights are significant, the study is not without limitations. For instance, the research results have not been validated in LUAD animal models yet. Additionally, the precise sites on SOX2 where WTAP performs m6A methylation remain elusive. Nevertheless, this work has illuminated the pivotal role of the WTAP/SOX2/Wnt/β-catenin axis in orchestrating the aggressive behaviors of LUAD cells. Interventions targeting the WTAP/SOX2/Wnt/β-catenin axis may represent a new avenue for the treatment of LUAD patients.

## Data Availability

The original contributions presented in the study are publicly available at https://doi.org/10.6084/m9.figshare.32346390.

## Supplementary material

SUPPLEMENTARY FIGURE S1 SOX2 knockdown attenuates LUAD malignancy. HCC827 cells were stratified into two groups: si-NC and si-SOX2. **(A)** Transfection efficiency was confirmed by qRT-PCR in each group; **(B)** Western blotting was used to detect the protein expression of SOX2. **(C)** Colony formation assays were used to evaluate cell proliferation in each group; **(D)** Migration and invasion were analyzed using Transwell assays in each group; **(E)** Apoptosis was detected by flow cytometry in each group. **(F)** Cell cycle was detected by flow cytometry in each group. *P < 0.05.supplementary-material.

SUPPLEMENTARY FIGURE S2
**(A)** qRT-PCR was conducted to assess the mRNA expression levels of β-catenin. *P < 0.05.supplementary-material.
