# Supplementary figures and images for: WTAP-mediated m6A methylation of SOX2 affects lung adenocarcinoma malignancy via Wnt/β-catenin pathway
Source: Front Genet. 2026 Jun 22;17:1756241. doi: 10.3389/fgene.2026.1756241 (PMC13333474; doi:10.3389/fgene.2026.1756241)

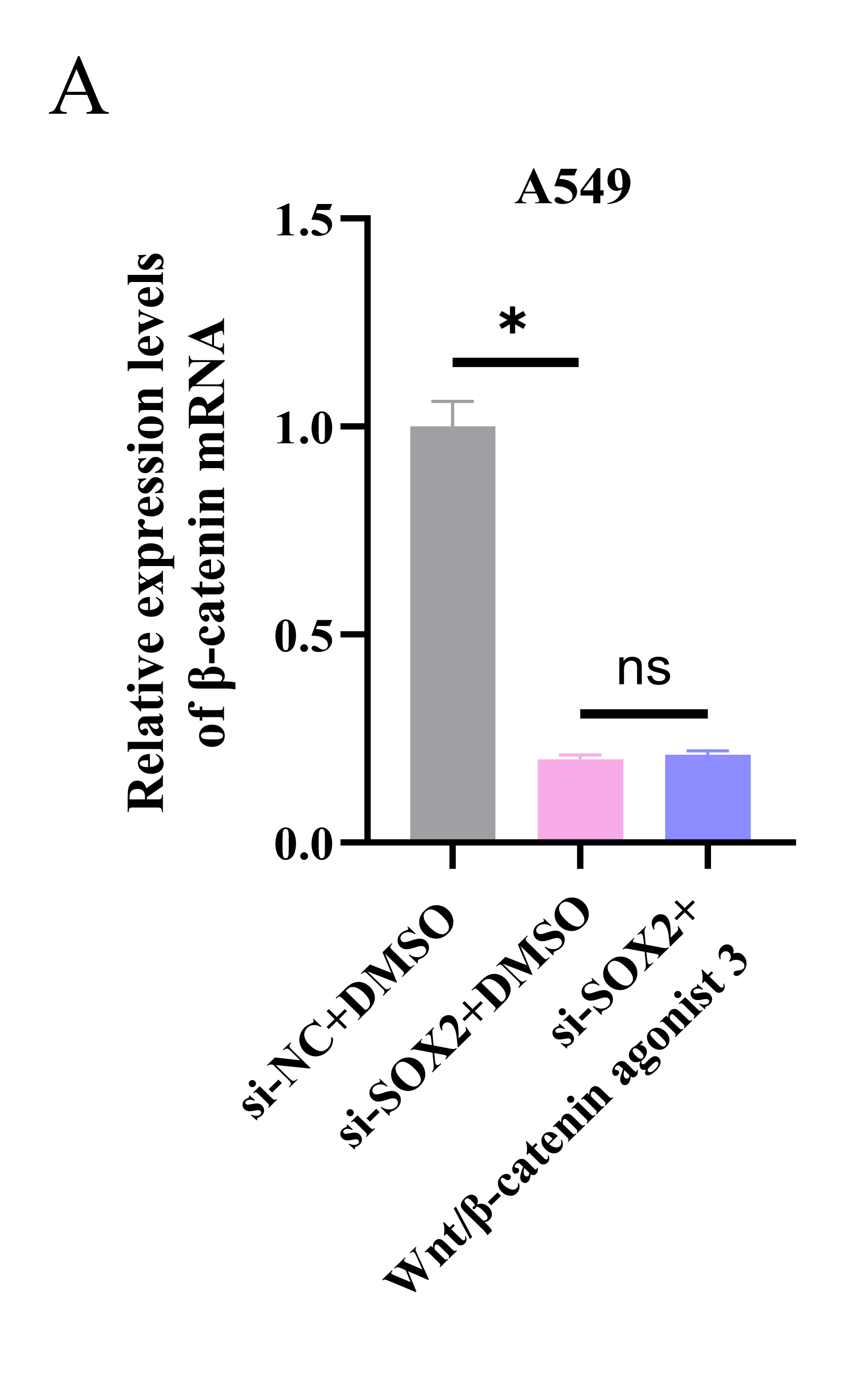

Supplement: Supplementary file 1 [file Image2.tif]

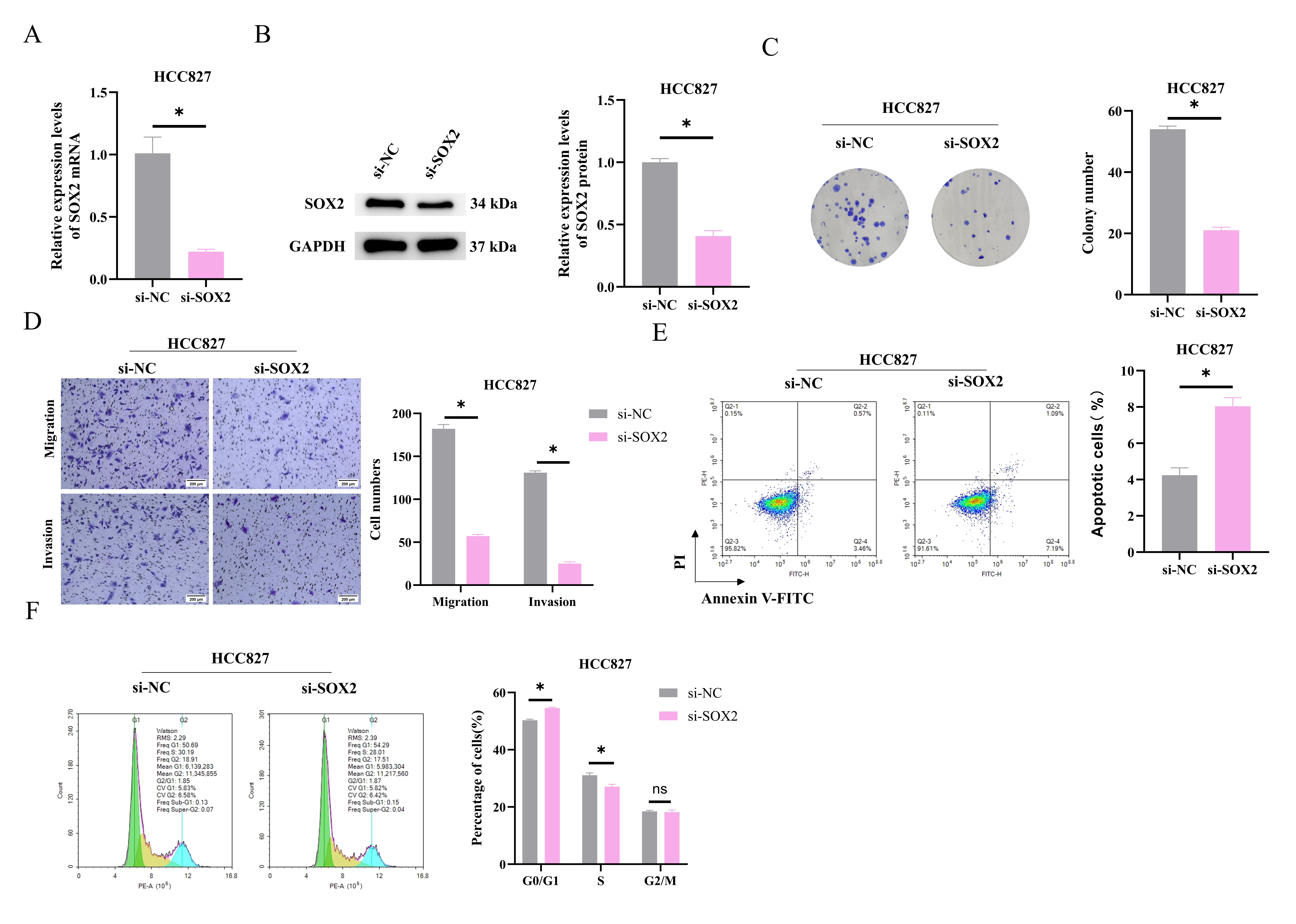

Supplement: Supplementary file 2 [file Image1.tif]
